# Small-Sized Reconfigurable Quadruped Robot With Multiple Sensory Feedback for Studying Adaptive and Versatile Behaviors

**DOI:** 10.3389/fnbot.2020.00014

**Published:** 2020-02-26

**Authors:** Tao Sun, Xiaofeng Xiong, Zhendong Dai, Poramate Manoonpong

**Affiliations:** ^1^Institute of Bio-inspired Structure and Surface Engineering, College of Mechanical and Electrical Engineering, Nanjing University of Aeronautics and Astronautics, Nanjing, China; ^2^Embodied AI & Neurorobotics Lab, SDU Biorobotics, Mærsk Mc-Kinney Møller Institute, University of Southern Denmark, Odense, Denmark

**Keywords:** quadruped robot, multiple sensory feedback, self-organized locomotion, vestibular reflexes, compliant control, flexible configuration

## Abstract

Self-organization of locomotion characterizes the feature of automatically spontaneous gait generation without preprogrammed limb movement coordination. To study this feature in quadruped locomotion, we propose here a new open-source, small-sized reconfigurable quadruped robot, called Lilibot, with multiple sensory feedback and its physical simulation. Lilibot was designed as a friendly quadrupedal platform with unique characteristics, including light weight, easy handling, modular components, and multiple real-time sensory feedback. Its modular components can be flexibly reconfigured to obtain features, such as different leg orientations for testing the effectiveness and generalization of self-organized locomotion control. Its multiple sensory feedback (i.e., joint angles, joint velocities, joint currents, joint voltages, and body inclination) can support vestibular reflexes and compliant control mechanisms for body posture stabilization and compliant behavior, respectively. To evaluate the performance of Lilibot, we implemented our developed adaptive neural controller on it. The experimental results demonstrated that Lilibot can autonomously and rapidly exhibit adaptive and versatile behaviors, including spontaneous self-organized locomotion (i.e., adaptive locomotion) under different leg orientations, body posture stabilization on a tiltable plane, and leg compliance for unexpected external load compensation. To this end, we successfully developed an open-source, friendly, small-sized, and lightweight quadruped robot with reconfigurable legs and multiple sensory feedback that can serve as a generic quadrupedal platform for research and education in the fields of locomotion, vestibular reflex-based, and compliant control.

## 1. Introduction

The motor behaviors of animals are characterized by numerous features (Dickinson et al., 2000). Several of these basic features, such as self-organization, vestibular reflexes, and compliance, play fundamental roles in achieving adaptive and versatile locomotion behaviors. Self-organization of locomotion represents the capability of autonomously spontaneous locomotion generation (Taga et al., 1991; Owaki et al., 2013; Tao et al., 2018). Vestibular reflexes and compliance can extend the functionality of self-organized locomotion in response to unexpected situations, such as abrupt changes in the ground plane and external perturbation. Therefore, understanding the biological principles of these properties contributes to revealing the underlying mechanisms of adaptive locomotion generation (Taga et al., 1991), and the subsequent development of advanced artificial legged robots (Hutter et al., 2017). However, it is not convenient to investigate the locomotor principles by means of animal experiments alone, because, in general, it is difficult to perform repeated measurements of the variables or quantities of unrestrained animal behaviors (Ijspeert, 2014). Fortunately, quadruped robots can serve as useful research tools for studying and validating the mechanisms or hypotheses of the various features of legged locomotion (Ijspeert, 2014; Karakasiliotis et al., 2016).

Over the past decades, several excellent quadruped robots have been developed for researching certain specific locomotion characteristics. For example, several large-sized quadruped robots, such as BigDog (Marc et al., 2008), LS3^1^, Wildcat^2^, and HyQ serial (Semini et al., 2011, 2016), with masses of over 100 kg and driven by hydraulics, have been developed through studies on high-power actuators, dynamic motions, and navigation (Raibert, 1986). The purpose of these studies focused on developing high-performance artificial machines for mobility in natural environments through engineering approaches. However, despite the performance of the robots shedding significant light on legged robotic applications in the transport field, thus far, they have not been used to investigate the mechanisms of self-organized locomotion generation and basic research. Moreover, their heavy weight and large size may result in a high-operation complexity as well as pose dangers for handlers or researchers who may use them as a legged platform for studying bio-inspired locomotion control (Eckert et al., 2018).

Therefore, several moderate-sized robots (with masses between 20 and 50 kg), such as the MIT Cheetah (Seok et al., 2013; Wensing et al., 2017; Bledt et al., 2018), ANYmal (Hutter et al., 2016), Spotmini^3^, and Laikago^4^, have been developed for researching the specific issue of quadrupedal locomotion, which includes proprioceptive actuators, electrically powered actuators, as well as learning agile and dynamic motor skills (Hwangbo et al., 2019). These robots have exhibited such stable and dynamic locomotor capabilities that they are quite suitable for studying high-level application techniques (for example, path planning, navigation, and transportation). However, it remains somewhat challenging to use these robots for investigating middle-level locomotion control (such as self-organized locomotion generation, reflex mechanisms, and compliant control), because their powerful actuators [that of the MIT Cheetah is ~230 Nm (Bledt et al., 2018)] still pose a danger to single researchers while directly manipulating their joints (Eckert et al., 2018). Furthermore, the development and hardware costs of these robots are quite high.

Consequently, small-sized, in detail, lightweight and compact, quadruped robots would offer an excellent option for studying adaptive locomotion generation. Several existing studies in this field have been presented to date. For example, Fukuoka et al. constructed a series of Tekken robots (Kimura and Fukuoka, 2004; Fukuoka and Kimura, 2009) and the Spinalbot robot (Fukui et al., 2019) to investigate bio-inspired adaptive locomotion mechanisms [central pattern generators (CPGs) and reflexes mechanism] with predefined interlimb coordination. Although their robots exhibit dynamic locomotion and gait transition, it is hard to use them for studying self-organized interlimb locomotion owing to their binary foot contact sensors, as the self-organized interlimb coordination is a continuous and dynamic interaction process among continuous sensory feedback, neural control, and body-environment dynamics (Owaki et al., 2013; Tao et al., 2018).

To overcome this problem, a series of the OSCILLEX robots (Owaki et al., 2012, 2013; Owaki and Ishiguro, 2017) were developed by Owaki et al. These robots were equipped with analog force sensors to obtain continuous foot contact feedback. They were used to investigate self-organized interlimb coordination for self-organized locomotion based on decoupled CPGs. With a simple robot structure in which each leg has two degrees of freedom (DOFs), OSCILLEX can autonomously perform adaptive locomotion patterns according to the walking speed and weight distribution. Nevertheless, it is difficult to use OSCILLEX with fixed leg configurations to investigate the effectiveness and generalization of self-organized locomotion regarding various leg configurations. Typically, existing small-sized quadruped robots lack sufficient sensory feedback (i.e., body inclination, joint current, and joint voltage) to investigate vestibular reflexes, compliance, and other adaptive and versatile behaviors in various expected situations. Moreover, they are not an open-source platform; therefore, limited access is offered to the community for rebuilding robots in their own studies. Therefore, to explore the features of quadrupedal locomotion (i.e., self-organized locomotion, vestibular reflexes, compliance, and their interactions, a small-sized, lightweight, and affordable quadruped robot) as a friendly research tool, with flexible configurations and sufficient sensory feedback, is a significant necessity for our research community.

In this study, we highlight our efforts to develop an open-source, small-sized, affordable quadruped robot, called Lilibot, in simulation and hardware, with flexibly reconfigurable leg orientations and multiple sensory feedback. The compact Lilibot was flexibly organized using lightweight modular components. These features enabled it to serve as a friendly quadrupedal platform. Furthermore, an adaptive neural controller was implemented to test Lilibot performance. The test included: (1) self-organized locomotion under flexibly reconfigurable leg orientations; (2) vestibular reflexes for stabilizing the body posture on a tiltable plane; and (3) compliant behaviors regarding an external load. Details on Lilibot and its adaptive neural control are provided in section 2. The performance examination of Lilibot is presented in section 3. Finally, section 4 provides a discussion of the experimental results and conclusion.

## 2. Methodology

In this section, we briefly introduce the main approaches and processes of Lilibot design, of which the basic restrictions are the small size, in detail, light weight, robust, and compactness but rich sensory feedback. To meet these requirements, selection and sizing of high-end small servo motors with comparative torque density were firstly considered. Secondly, according to the motor dimensions (XM430 from ROBOTIS^5^) and a template model (Full and Koditschek, 1999) of a mammal (i.e., dog), we determined the kinematics and link structures of the leg. The leg should have a large workspace for flexible leg configurations, as well as sufficient proprioceptive sensory feedback for compliant control. Thirdly, the legs were appropriately organized using a trunk, in which several necessary electrical devices for supporting the vestibular reflex control were installed. The final step was to optimize the mechanics of Lilibot iteratively through physical simulation controlled by specific algorithms in the virtual robot experimentation platform (V-REP) (Rohmer et al., 2013).

### 2.1. System Overview

**Figure 1:**
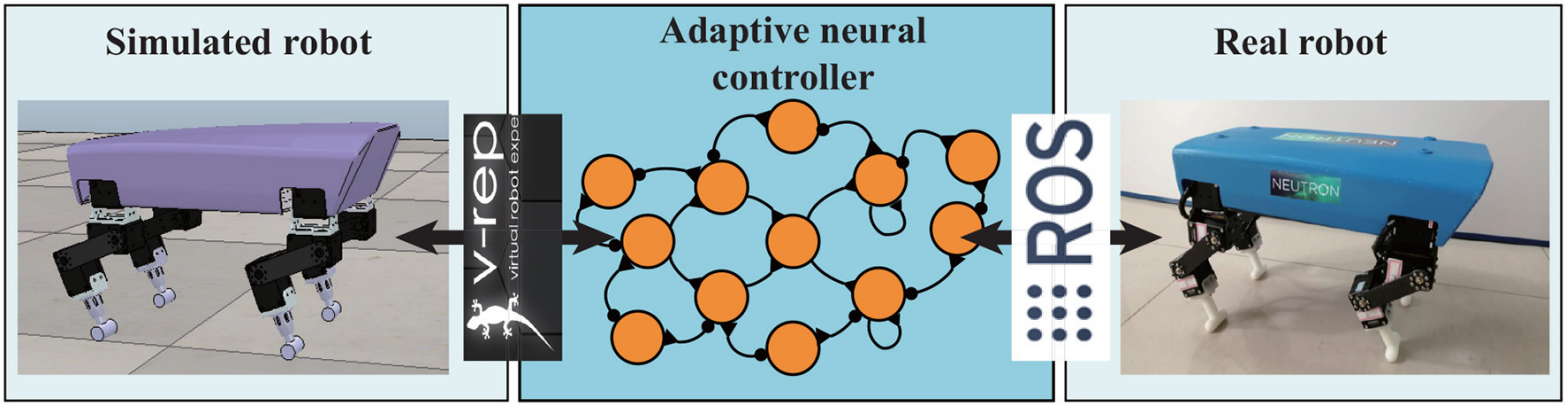
System overview of Lilibot. The adaptive neural controller is implemented in ROS such that it can directly communicate with both the simulated robot in the V-REP simulation and the real robot. The simulated and real robots were consistently developed, such that the simulation demonstrates a good estimation of the actual performance. A video showing a comparison between the simulated and real robot behaviors can be seen at http://www.manoonpong.com/Lilibot/video6.mp4.

**Figure 2:**
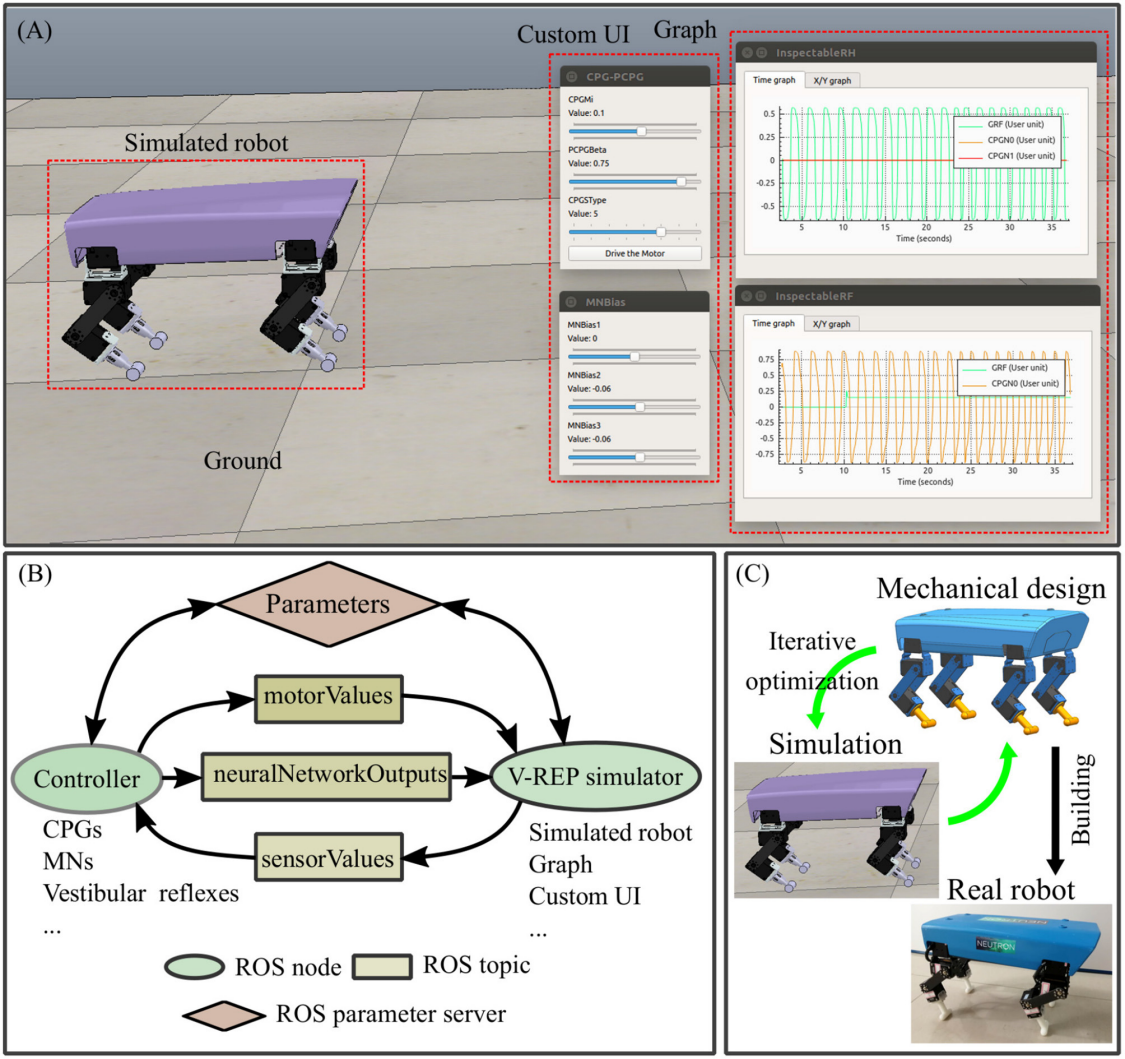
Lilibot simulation and its developmental process. **(A)** The virtual robot experimentation platform (V-REP) (Rohmer et al., 2013) simulation scene of Lilibot. The mechanical model of Lilibot is loaded into the V-REP to create a simulated robot in a virtual environment. The V-REP provides the graph tools to monitor various signals (including motor commands, outputs of the sub-control modules, and sensory signals). Besides, the custom UI of the V-REP can be used to adjust the control parameters [such as in this study, CPG frequency and amplitude as well as sensory feedback strength of the decoupled CPGs control (see Tao et al., 2018) and the weight parameters of the vestibular reflex control (Figure 7)]. **(B)** The simulator is based on the V-REP and the robot operation system (ROS) (Quigley et al., 2009). The communication between the controller (ROS node1) and the simulator (ROS node2) is accomplished through three ROS topics and a parameter server. The topics include (1) a “motorValues” topic transmitting motor commands of joints from the controller to the simulated robot; (2) a “sensorValues” topic transmitting sensory signals of the simulated robot to the controller; (3) a “neuralNetworkOutputs” topic transmitting the outputs of the sub-control modules. The parameters of the controller are accessed through a ROS parameter server. The communication between the controller (ROS node1) and the real robot (ROS node3) is also performed in the same manner through the ROS topics and the parameter server. **(C)** The mechanical design is iteratively optimized using the V-REP simulation.

In this quadrupedal locomotion research system, the real Lilibot and its simulated model in the V-REP are controlled by an adaptive neural controller (Figure 1) through the Robot Operation System (ROS) (Quigley et al., 2009). The ROS serves as a framework for linking the three components (simulated robot, controller, and real robot) and providing their communication channels through the ROS interfaces. In the simulation (Figure 2A), various values [i.e., motor commands from the controller, sensory signals from the simulated robot, and outputs of all sub-control modules (Figure 2B)] of the system can easily be monitored using the graph tools of the V-REP. The parameters of the monitored values can be easily adjusted through the user interface (UI) of the V-REP. Moreover, kinematics and dynamics modules as well as scene objects (i.e., force sensors) of the V-REP can be used to inspect the leg workspace and joint forces of Lilibot. The measurements can be regarded as estimations to iteratively optimize the leg structure design before constructing a physical one (Figure 2C). From this point of view, we can improve the robot development efficiency and save the development cost. In the Lilibot system (Figure 1), we can first develop and evaluate an adaptive neural controller in the simulation and then directly test it on the real robot without any modifications. The details of the real Lilibot and the adaptive neural controller are presented in the following parts.

### 2.2. Robot Development

#### 2.2.1. Specifications of Lilibot

**Figure 3:**
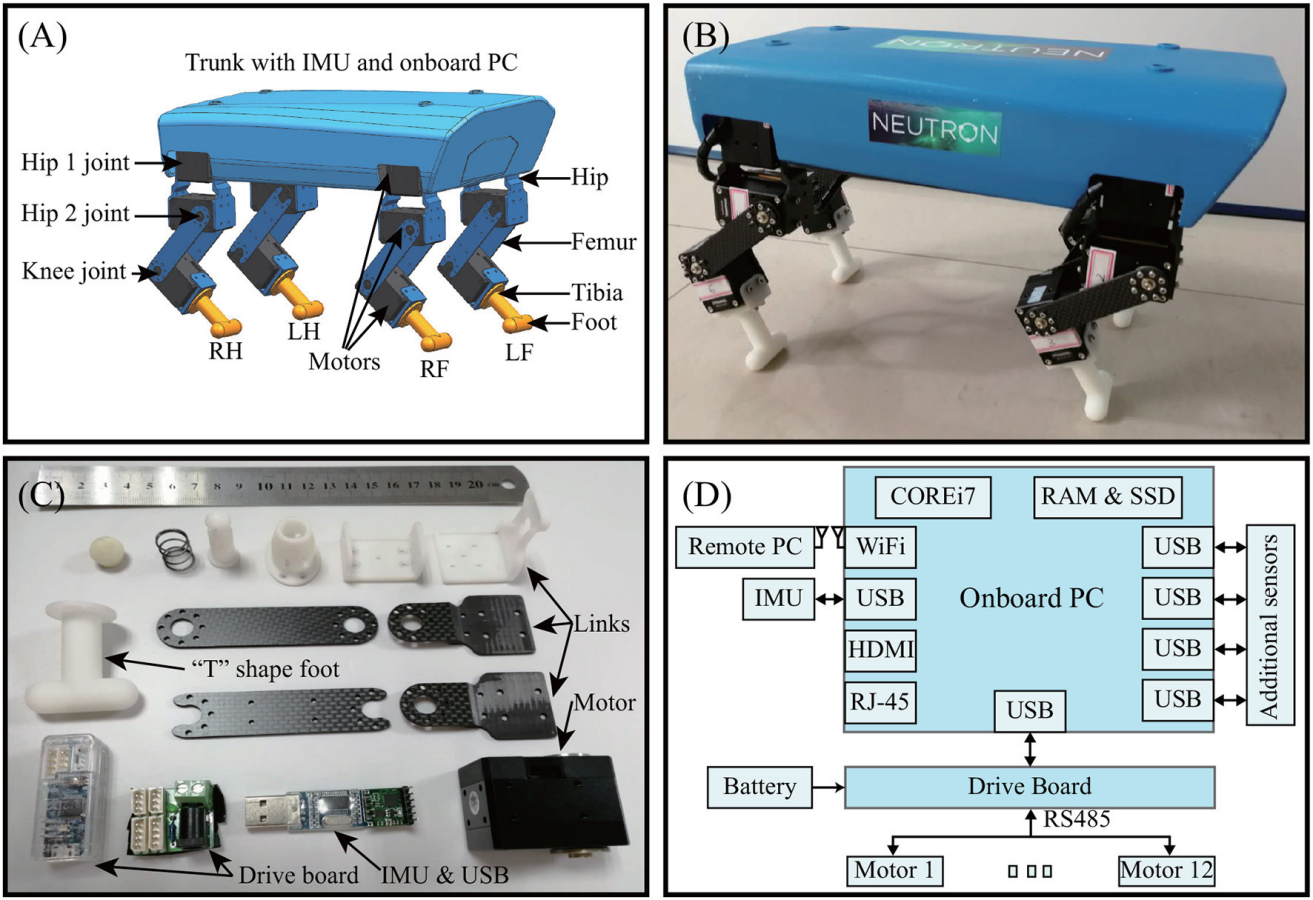
Lilibot. **(A)** CAD model. **(B)** Real robot with a weight of 2.5 kg. Its length, width, and height are 30, 17.5, and 20 cm, respectively, when it stands. **(C)** Main components of one leg. **(D)** Mobile processor system.

The final version of Lilibot, following optimization by means of simulation in the V-REP, is presented in Figure 3. With reference to the current proficient template [SLIP (Poulakakis and Grizzle, 2009; Yu et al., 2012)] and anchor (for example, Spotmin and Laikago) of quadrupedal locomotion, Lilibot was designed with four identical legs, namely the right front (RF) leg, right hind (RH) leg, left front (LF) leg, and left hind (LH) leg. Each leg consists of three links, namely the hip, femur, and tibia, and has three active joints (hip 1 joint, hip 2 joint, and knee joint), which are driven by smart servo motors (4.2 Nm, XM430 from ROBOTIS). The tibia link is connected to a foot with a shape resembling a “T” that provides a large support area. The main components of the leg are illustrated in Figure 3C, and are constructed using 3D printing or made of carbon fiber. The four legs are attached to a rigid trunk that carries an inertial measurement unit (IMU), an onboard PC, and a Li-ion battery (14.8 V–4 Ah), which could supply Lilibot as a compact mobile platform to run for more than an hour. With a payload of ~1.25 kg, Lilibot can walk for up to 30 min^6^. The weight and dimensions of Lilibot are presented in Table 1. Detailed information regarding the leg configurations and multiple sensory feedback is provided in the following subsection. The open source (including the code for the interface and 3D CAD model) of Lilibot can be viewed at https://gitlab.com/neutron-nuaa/lilibot. The total hardware cost of Lilibot is 5,381 USD.

**Table 1:** The weight and dimensions of Lilibot.

Weight	2.5 kg
Lengtd	30 cm
Widtd	17.5 cm
Height	20 cm

#### 2.2.2. Flexibly Reconfigurable Leg Orientations

**Figure 4:**
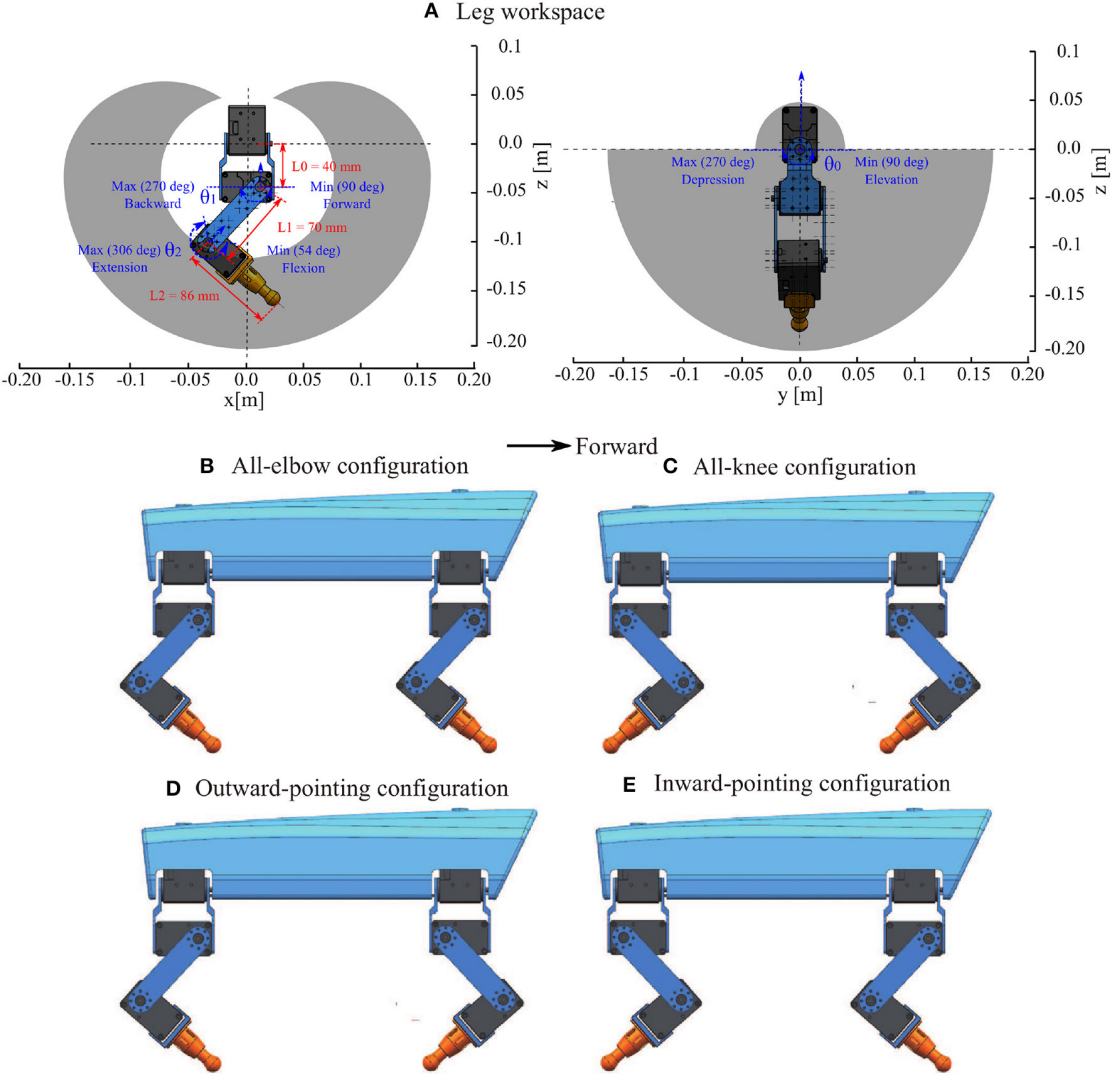
Leg workspace and various configurations. **(A)** The leg has a large and symmetric workspace that enables the robot to exhibit four different configurations or orientations, as indicated in **(B–E)**. **(B)** All-elbow configuration. **(C)** All-knee configuration. **(D)** Outward-pointing configuration. **(E)** Inward-pointing configuration.

Different species of four-limbed mammals, such as dog, infant, and horse, particularly with varying size scales, have distinct skeleton topologies, especially in the legs. Therefore, when researchers have modeled their structures for building real robots (anchor models) to study quadrupedal locomotion, various leg orientations (joint/leg configurations) have appeared in certain impressive robots (Marc et al., 2008; Fukuoka and Kimura, 2009; Semini et al., 2011; Sprowitz et al., 2013; Wensing et al., 2017; Bledt et al., 2018). Several researchers have specifically studied the influence of multiple leg orientations on the movement performance. For instance, Xiuli et al. demonstrated that centrosymmetric joint configurations (i.e., outward and inward pointing, Figure 4) are beneficial for avoiding slipping to increase stability (Xiuli et al., 2005). Moreover, Meek et al. argued that appropriate leg configurations could achieve optimal stabilization in specific situations, such as a simulated quadruped robot with inward-pointing configuration has the lowest pitching motion compared to other three configuration types (Meek et al., 2008). Therefore, it is necessary to construct Lilibot with flexible leg configurations, thereby facilitating studies on the effectiveness and generalization of locomotion control under the different configurations.

Based on the assumptions, we developed Lilibot with flexible leg configurations. This advantage results from each joint of the legs having extensive rotation ranges, which provide the legs with a large and symmetric workspace (Figure 4A). Hence, Lilibot can flexibly reconfigure its leg orientations. Figures 4B–E present Lilibot with four configuration types using different leg orientations. With reference to Xiuli et al. (2005), we called these the all-elbow, all-knee, outward-pointing, and inward-pointing configurations. The configurations have been used in various classical robots; for example, the all-elbow and all-knee configurations were applied to certain small- and moderated-sized robots [Spotmini, Laikago, and MIT cheetah (Seok et al., 2013)], while the inward-pointing configuration was applied to several large-sized quadruped robots [including HyQ (Semini et al., 2011) and BigDog (Marc et al., 2008)], and the outward-pointing configuration was applied to the very heavy robot LS3.

#### 2.2.3. Multiple Sensory Feedback

Abundant sensory feedback plays a vital role in the successful implementation of various control strategies in robots. Thus, we installed as many sensors as possible on this relatively small robot to investigate adaptive and versatile behaviors (see Table 2). As a result, a nine-axis IMU and twelve smart actuators with encoders and analog-to-digital converters were installed in Lilibot. The IMU (JY901 of ZNJ) can measure the body inclination, angular velocities, and velocities around three axes. Moreover, each actuator with an encoder and one analog-to-digital converters on the joint can detect and feed the joint position, velocity, current, and voltage. Furthermore, considering the simplification of the foot structure, we utilized the current feedback of the servo motors at the knee joints to reflect the ground reaction force (GRF) quantity by means of an indirect conversion algorithm. The mechanism of the algorithm is that the GRF of a leg, which indicates the load on the leg, has a positive correlation with the keen joint current. The algorithm is given by the following equations:

(1) $$\begin{array}{l} f_{i}=\{\begin{array}{l} 0,0\geq g_{i}\\ g_{i},0<g_{i}<f_{\text{limit}}\\ f_{\text{limit}},g_{i}\geq f_{\text{limit}} \end{array}\;,f_{\text{limit}}=1.2, \end{array}$$

(2) $$\begin{array}{l} g_{i}=k_{i}\tau_{i}+b_{i}\left(v\right), \end{array}$$

(3) $$\begin{array}{l} k_{i}=\{\begin{array}{l} 1.1,i=0,1\\ -1.1,i=2,3 \end{array}, \end{array}$$

(4) $$\begin{array}{l} b_{i}\left(v\right)=\{\begin{array}{l} -0.3+1.2v,i=0,3\\ -0.25+1.2v,i=1,2 \end{array}, \end{array}$$

where *f*_*i*_ represents the indirect GRF of the leg *i*, which is normalized into a range (0, 1). τ_*i*_ is the current feedback of the servo motor at the knee joint, while *k*_*i*_ and *b*_*i*_ are the slope and intercept of the linear function *g*_*i*_, respectively. *f*_limit_ is the threshold of the indirect GRF, and *v* is the joint velocity. A measured GRF (obtained from the custom-designed force plate platform for legged robots) is used as a baseline for tuning the model parameters. One can observe a positive correlation between the keen joint current signal and the GRF signal. The signals show high activation (>0.0) when the leg is in a stance phase and low activation (around 0.0) when it is in a swing phase. An experiment for tuning the parameters of the model can be seen in Figure S1. This algorithm not only decreases the robot structural complexity, but also increases the stability of the perceptive system of Lilibot, owing to removing the extra force sensors on its legs and, hence, reducing complex signals acquisition and communication tasks.

**Table 2:** All sensors and amount of sensory feedback of Lilibot.

**Sensors**	**Feedback**	**Quantity/61**
IMU	Body inclinations	3
	Angular velocities	3
	Velocities	3
Encoder	Joint positions	12
	Joint velocities	12
AD	Joint currents	12
	Joint voltages	12
Indirection measurement	Foot contact force	4

Although Lilibot exhibited a small size and compact space, the onboard PC (NUC7 from Intel Inc.) can simultaneously acquire 61 sensory feedback signals (see Table 1) at a frequency of 180 Hz. The rich sensory feedback and compact actuators enable Lilibot to be a compact and generic legged platform for supporting various control modes (e.g., position control, velocity control, and compliant control, as well as vestibular reflex control). In addition to the existing sensors, additional USB ports of the onboard PC provide available interfaces for including other sensors.

### 2.3. Adaptive Neural Controller

**Figure 5:**
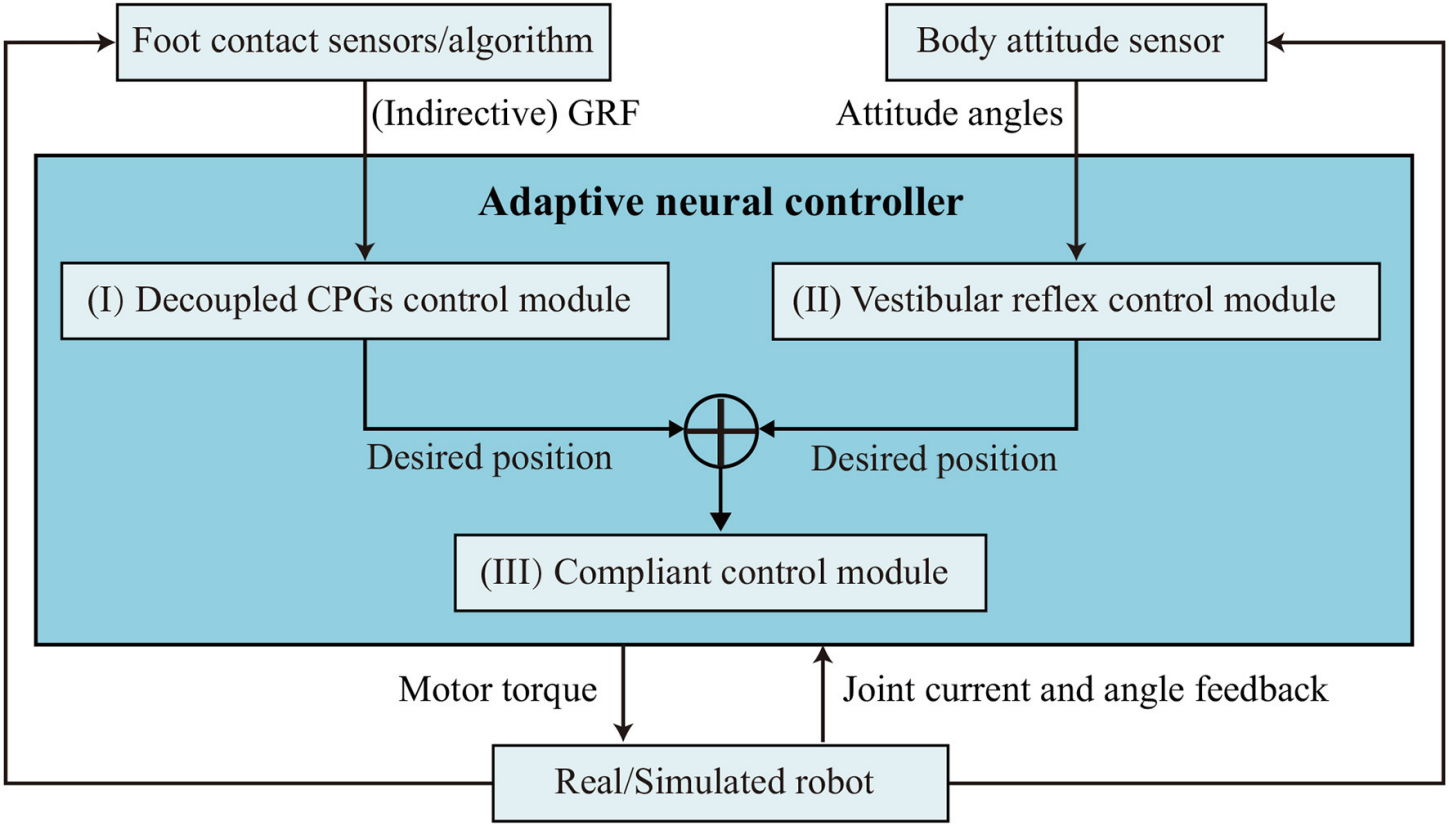
Framework of adaptive neural controller.

To test the performance of Lilibot as a friendly quadrupedal platform, particularly for studying adaptive and versatile behaviors, including vestibular reflexes and leg compliance, it is necessary to implement control. For this purpose, by exploring bio-inspired approaches with sensorimotor loop (Hülse et al., 2007) and referring to Owaki et al. (2013), an adaptive neural controller (Figure 5) was developed^7^. It consists of three sub-control modules: (I) decoupled CPGs control; (II) vestibular reflex control; and (III) compliant control. The decoupled CPGs control can be used to validate whether Lilibot could perform self-organized locomotion, derived from the self-organized interlimb coordination, as well as its effectiveness under different leg orientations. The vestibular reflex control was designed to validate whether Lilibot could adaptively stabilize the body posture on a tiltable plane. The compliant control based on the hybrid torque-position control principle was designed to test whether Lilibot could exhibit compliant behaviors when responding to an external load. Both the decoupled CPGs and vestibular reflex control modules output the desired positions of all joints. The desired positions are transmitted to the compliant control module (low-level control). Thereafter, the compliant control transforms the desired positions into the desired currents that finally drive the robot as torque control.

#### 2.3.1. Decoupled CPGs Control

**Figure 6:**
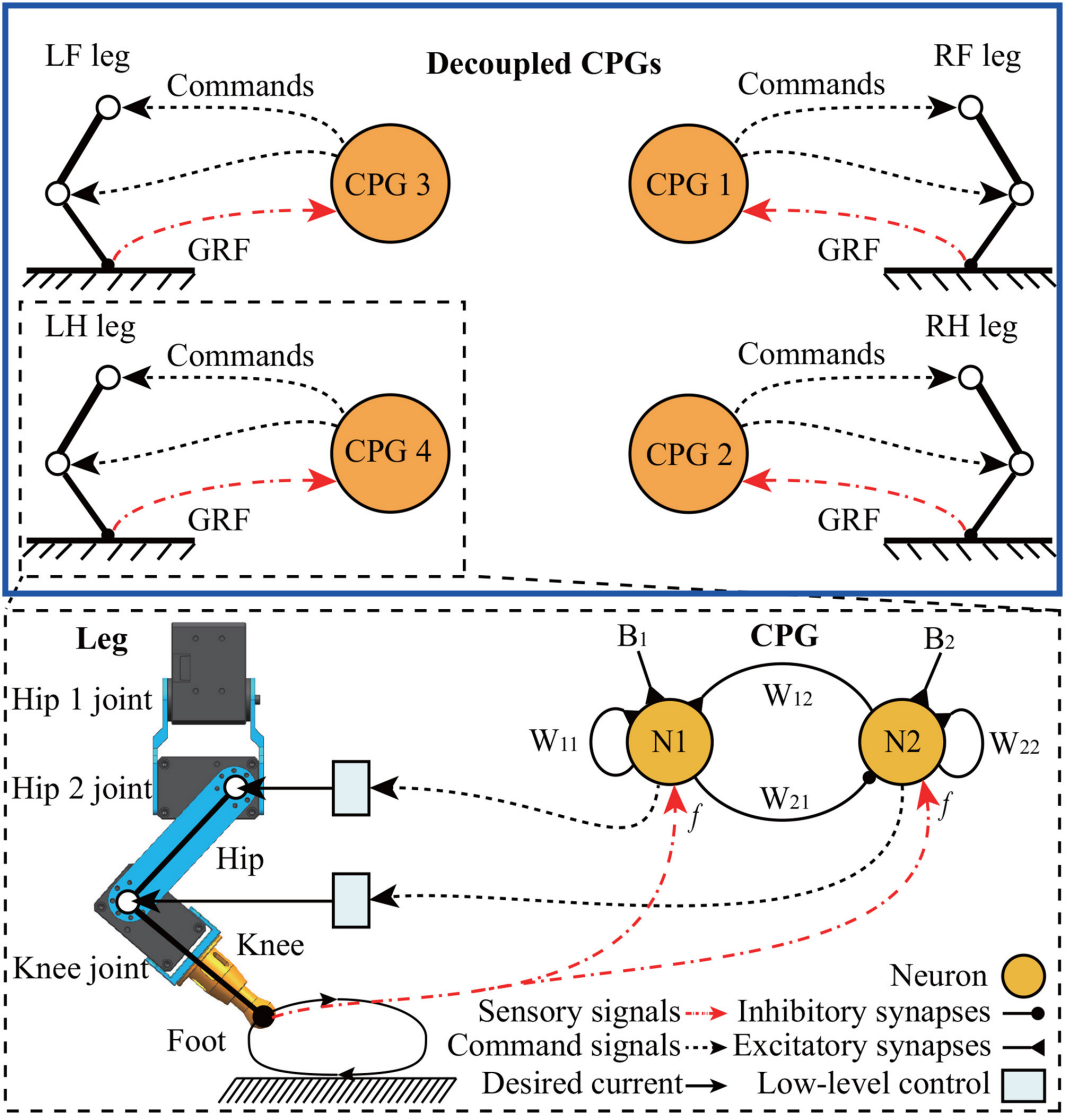
Schematic of decoupled CPGs. Each CPG, which comprises two mutually interactive neurons, obtains a global robot state through the GRF as sensory feedback. The mathematical model of the decoupled CPGs can be seen in the Supplementary Material. The weights and bias terms of the CPG were empirically set to *W*_12_ = 0.21, *W*_21_ = −0.21, *W*_11_ = 1.4, *W*_22_ = 1.4, and *B*_1, 2_ = 0.01 in the following experiments. The details of the parameter setup can be found in Manoonpong et al. (2008).

The details of the decoupled CPGs control are illustrated in Figure 6. The control has four identical and decoupled neural SO(2) oscillators (Pasemann et al., 2003) (acting as CPGs). A single leg of Lilibot is controlled by the decoupled CPG consisting of two fully connected standard additive time-discrete neurons, N1 and N2, both using a sigmoid transfer function. Although there is no connection between the CPGs, their outputs interact through their corresponding/local foot contact feedback, i.e., GRFs. The GRFs shape the outputs of the CPGs such that proper phases between the CPGs emerge to obtain a stable gait. The two outputs with a phase shift of π/2 are transmitted to control the actuators of the hip 2 and knee joints of the leg (Figure 3). As a result, the two joints of each leg move with a phase shift of π/2. In this manner, for each leg, the knee joint flexes first and is followed by the hip 2 joints generating forward leg motion in the swing phase. During the stance phase, the knee joint extends to allow the foot to touch the ground before the hip 2 joint moves backward. Note that the hip 1 joints of all legs are set to fixed positions for the sake of simplicity. This intralimb movement coordination guarantees ground clearance during the swing phase and ground contact during the stance phase.

To achieve stable gaits, a self-organized method is applied by means of physical communication based on local sensory feedback (namely, GRF) (Tao et al., 2018). In this manner, the GRFs are fed to the corresponding CPGs to modulate their phases. Owing to the GRF differences among the four legs when the robot wriggles on the ground, the effectiveness of the modulations is diverse, and thereby, the phase shifts among the four CPGs emerged autonomously. This results in phase differences in the limb movements. As the phase differences converge, a self-organized locomotion gait is generated.

#### 2.3.2. Vestibular Reflex Control

**Figure 7:**
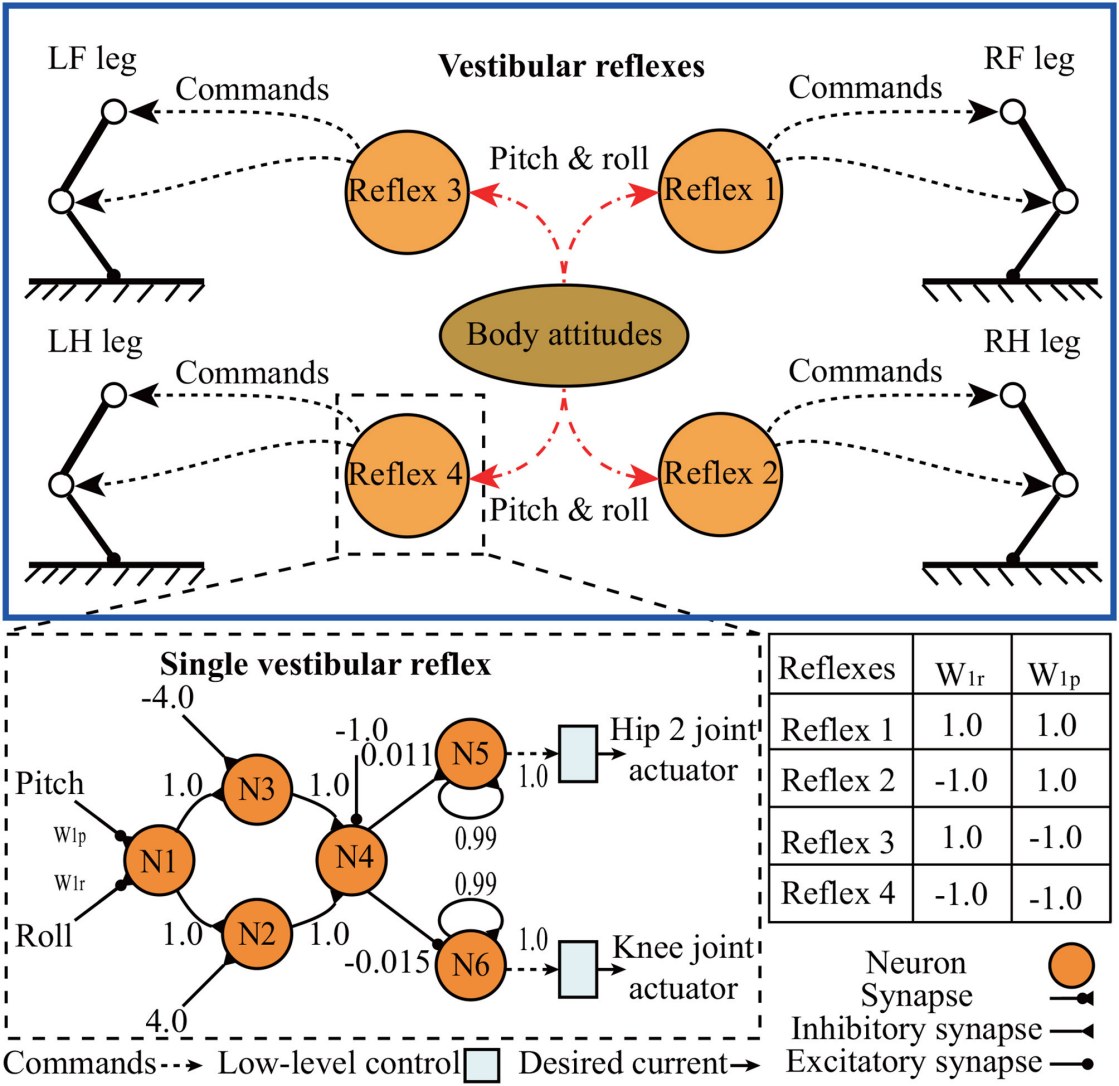
Schematic of vestibular reflex control mechanism. The weights of the neural reflex network are set empirically.

Inspired by natural vestibular reflex behaviors, our neural reflex mechanism (Tao et al., 2018) was extended to vestibular reflexes for testing the performance of the IMU inclination measurement on Lilibot, as well as the capability of Lilibot to stabilize its body posture. In this case, four distributed vestibular reflexes (Figure 7) were implemented to control the legs depending on the body pitch and roll inclination. For example, when there is a detected inclination in the pitch or roll plane, the downward-inclined and upward-inclined legs would be controlled to extend and flex, respectively.

The single vestibular reflex is realized by a feedforward neural network with four layers composed of six neurons. Their transfer functions are hyperbolic tangent functions, except for those of N5 and N6, which are linear functions. The weights w_1r_ and w_1p_, are specified in the table in Figure 7, and determine the interlimb coordination of the responding movements. Although the neural network has non-linear transforms, for the sake of simplification, the functionality of the transformation can be considered as a combination of several multiplication and addition operations because the inputs (body inclination) of the neural network are scaled into the linear interval of the transfer functions. The neural network outputs two coordinative signals, which are transmitted to the hip 2 and knee joints of a leg through low-level control (e.g., compliant control), thereby manipulating the leg to extend or flex depending on the body inclination.

#### 2.3.3. Compliant Control

**Figure 8:**
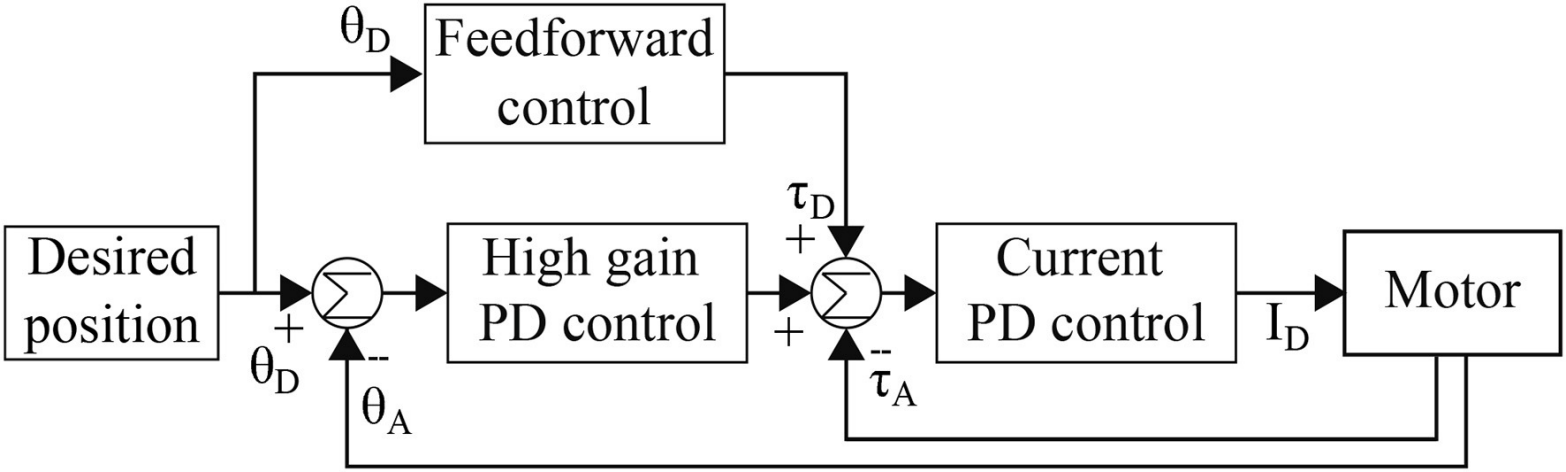
Block diagram of compliant control mechanism loop: θ_D_ and θ_A_ represent the desired and actual joint positions, respectively; τ_D_ and τ_A_ are the desired and actual motor torques; and I_D_ is the desired current for driving the motor.

As a low-level control, compliant control (Figure 8) is implemented to control actuators precisely and gently when the robot encounters unexpected external perturbation. It has three control loops: (1) feedforward control for rapid response to the desired position, (2) high gain proportional derivative (PD) control for position control with feedback to reduce the position error, and (3) current PD control for torque control. The outputs of the position control are the desired inputs of the torque control. The control framework was implemented on Lilibot to demonstrate compliance for negotiating external loads, as detailed in subsection 3.3.

## 3. Experiments and Results

Four sets of experiments were performed to test the performance of Lilibot, implemented with the presented adaptive neural controller, as a quadrupedal platform. The three control modules (decoupled CPGs control, compliant control, and vestibular reflex control) in the adaptive neural controller were conducted separately first for clearly demonstrating the functionality of the different features of Lilibot. Subsequently, a combination of the vestibular reflex and compliant controls was executed to evaluate their integrated functions. Therefore, the experiments consisted of: (1) self-organized locomotion under different leg orientations, driven by the decoupled CPGs control, (2) leg compliance to compensate for an unexpected external load, driven by the compliant control, (3) body stabilization on a tiltable plane, driven by the vestibular reflex control, and (4) body stabilization and payload compensation on a tiltable plane, driven by the combination of the vestibular reflex and compliant controls.

### 3.1. Self-Organized Locomotion Under Different Leg Orientations

**Figure 9:**
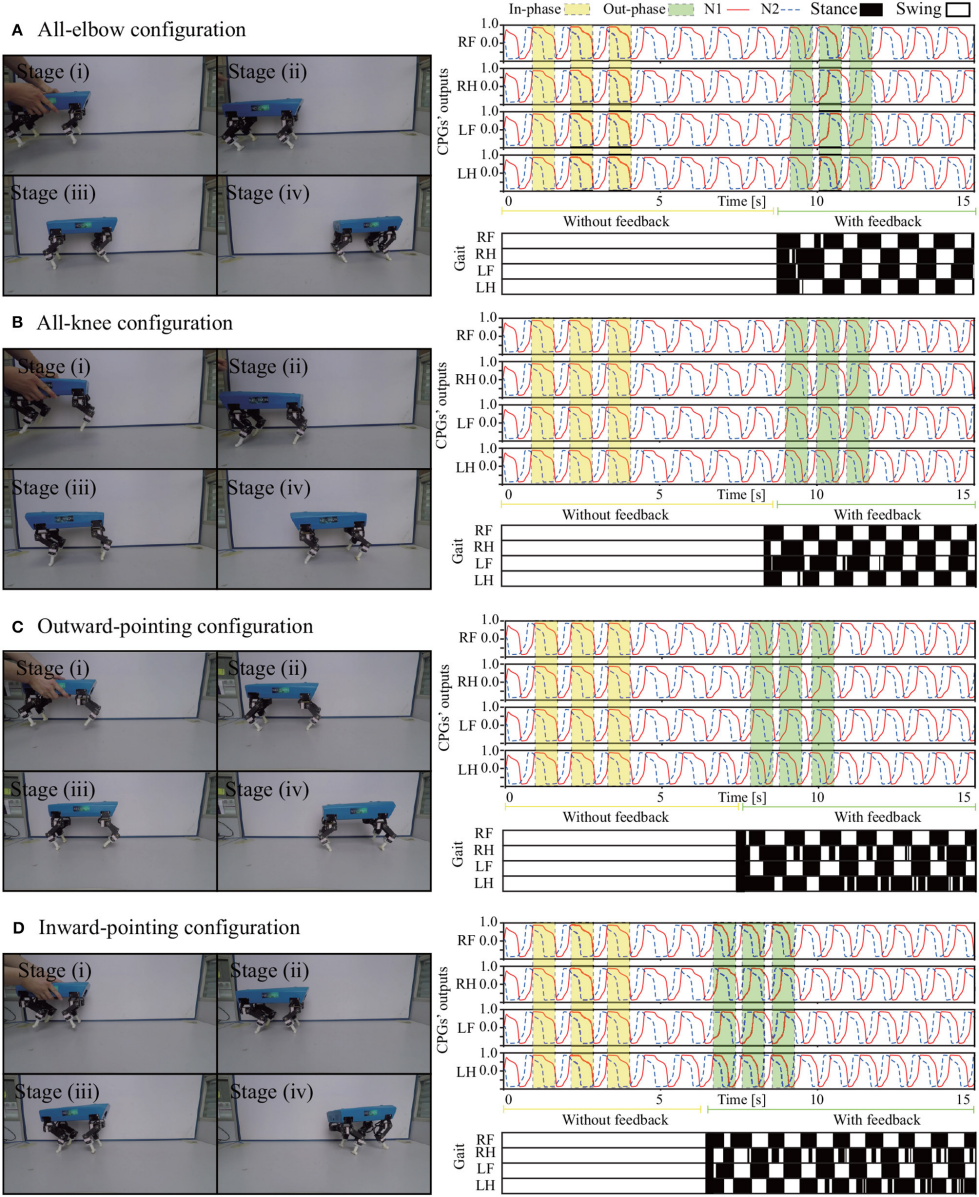
Process of self-organized locomotion generation under four leg orientation types, as indicated in **(A–D)**. **(A)** All-elbow configuration. **(B)** All-knee configuration. **(C)** Outward-pointing configuration. **(D)** Inward-pointing configuration. The outputs of the CPGs started in phase, and once the robot interacted with the ground, the phases began to be adjusted by the GRFs. The gaits quickly emerged within 8 s.

Four experiments were performed to test whether Lilibot could exhibit self-organized locomotion driven by the decoupled CPGs control under the four leg orientation types (four leg configurations (see Figures 4B–E). In all experiments, the decoupled CPGs were initialized to output in phase with the same parameters, while the robot was held in the air at the beginning [see the stage (i) in Figure 9]. We observed that as soon as the robot was placed on the ground [see stage (ii) in Figure 9], the representation feedback of the GRFs on the feet was fed to the CPGs to modulate their neural activities, thereby adapting the phases of the CPGs' outputs [see stage (iii) in Figure 9]. Consequently, a trot gait autonomously emerged in stage (iv). In the gait diagram (see Figure 9), the black regions represent the stance phases, which are detected by the GRFs. For example, if a GRF is higher than a threshold value, a stance phase is indicated. Thin stripes in the gait diagram represent oscillations of the GRFs data around the threshold value. According to the results, such a quadruped-like gait was generated in a self-organized manner under the four leg orientation types when using our decoupled CPGs. A video clip of this experiment was recorded (at http://www.manoonpong.com/Lilibot/video1.mp4).

To evaluate the energetic cost of the locomotion under the four leg configurations, the specific resistance was used. It is defined as the ratio between the consumed energy and the transferred gross weight times the distance traveled (Manoonpong et al., 2016):

(5) $$\begin{array}{l} \epsilon =\frac{E}{mgd}, \end{array}$$

where *E* is the consumed energy of the robot motors when the robot walks a distance *d* (i.e., 1 m) and *mg* is the weight of the robot. The energy is estimated from: *E* = *IVt*, where *I* and *V* are the electric current and voltage, respectively. They can be acquired from the joint current and voltage sensors. *t* is the time the robot uses when it walks a distance *d*. The average specific resistances of Lilibot under the four leg configurations (all-elbow, all-knee, outward-pointing, and inward-pointing) are ~3.57±0.12, 3.32±0.43, 5.16±0.32, and 3.82±0.30, respectively. A low ϵ corresponds to high energy-efficient walking. Thus, the results indicate that the all-elbow and all-knee configurations have relative high energy efficiency and the outward-pointing configuration exhibits the lowest energy efficiency. The details of the experiment can be seen in Figure S2.

### 3.2. Compliant Behavior for Unexpected Load Compensation

**Figure 10:**
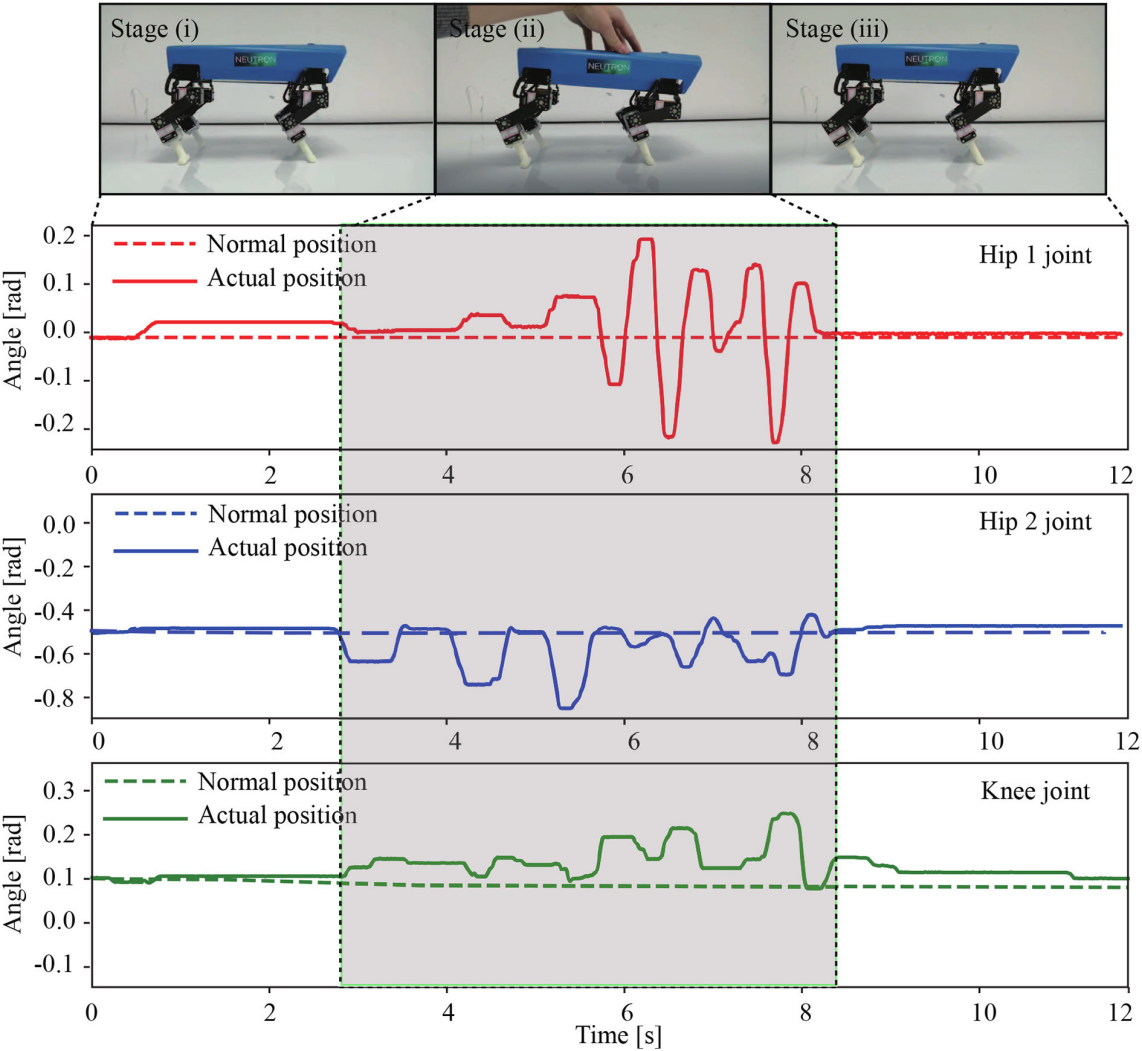
Compliant behavior of Lilibot and angle feedback of the hip 1, hip 2, and knee joints of the RF leg. The normal positions of joints were the desired positions of compliant control. The robot was placed on the ground and was stooding in the initial stage (i). In stage (ii), a hand was used to apply a force on its body, and the robot exhibited compliance to compensate for the perturbation. During stage (iii), the robot returned to its normal position after the load was removed.

Compliance is an important function that allows a robot to effectively deal with unexpected load or large perturbation. In this experiment, we demonstrated that Lilibot could deal with an unexpected load (i.e., hand loading) based on the presented compliant control (Figure 10). To clearly demonstrate the effect of the compliant control, we switched off the decoupled CPGs and vestibular reflex control (high-level control) by setting their outputs to zeros (see Figure 5). At the beginning of the experiment, the robot stood on the ground in stage (i), in which all joints stayed in their normal positions. The normal positions as a reference were inputted into the compliant control as the desired positions (see Figure 8). Thereafter, we pushed the robot body by a hand in stage (ii) from ~3 to 8.2 s, and instead of the rigid status controlled only by highly stiff position control, the robot actively exhibited softness. When the push was withdrawn in stage (iii), the robot returned to its initial standing posture. As an example, the angle feedback of the right front leg joints is depicted in Figure 10, reflecting the active compliant movement of the joints responding to the external hand load. Consequently, it was concluded that Lilibot is capable of exhibiting compliant leg behavior based on our controller. A video clip of this experiment was recorded (at http://www.manoonpong.com/Lilibot/video2.mp4).

### 3.3. Body Stabilization on a Tiltable Plane

**Figure 11:**
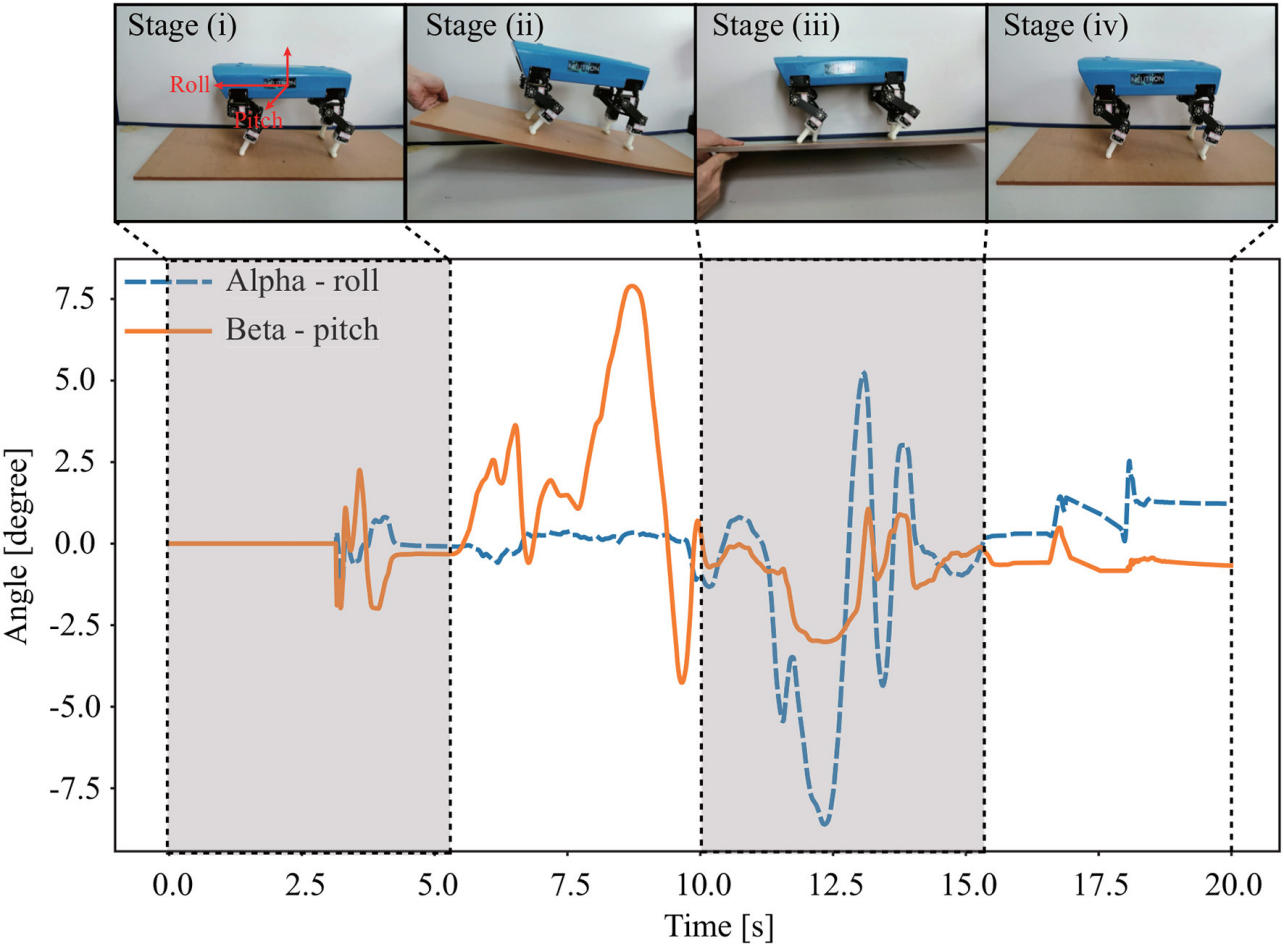
Snapshot and body attitude angles of Lilibot in the experiment, where Lilibot sustained its body attitude stabilization while the supported tiltable plane inclined around the pitch and roll planes in stages (ii) and (iii), respectively.

To test the effectiveness of the IMU sensor of Lilibot for body stabilization, an experiment was conducted using the presented vestibular reflexes on Lilibot because the vestibular reflex control can stabilize the robot according to the inclination feedback measured by the IMU. Firstly, Lilibot, with vestibular reflexes, was placed on a tiltable plane (see Figure 11). The experiment consists of four procedures [stages (i)–(iv)]. The plane pitch angle was changed in stage (ii), and the robot performed extension or flexion of the legs to stabilize the body, depending on the inclination feedback from the IMU. As a result of the vestibular reflexes, the pitch angles of the body returned to ~0 following oscillation. Similarly, the changed plane roll angle made the robot extend or flex its legs to maintain its body level in the roll direction during stage (iii). The experimental results demonstrate that the vestibular reflexes could sustain the stabilization of Lilibot on a tiltable plane. Therefore, we also assert that the IMU enabled Lilibot to exhibit vestibular reflexes. A video clip of this experiment was recorded (at http://www.manoonpong.com/Lilibot/video3.mp4).

### 3.4. Body Stabilization and Payload Compensation on a Tiltable Plane

A combination among the self-organized locomotion, vestibular reflexes and leg compliance plays a crucial role for adaptive locomotion on natural terrains (Fukuoka et al., 2003; Liu et al., 2013). As an example here, we show a combination of the vestibular reflexes and the leg compliance. This combination was applied to demonstrate body stabilization under a complex situation.

**Figure 12:**
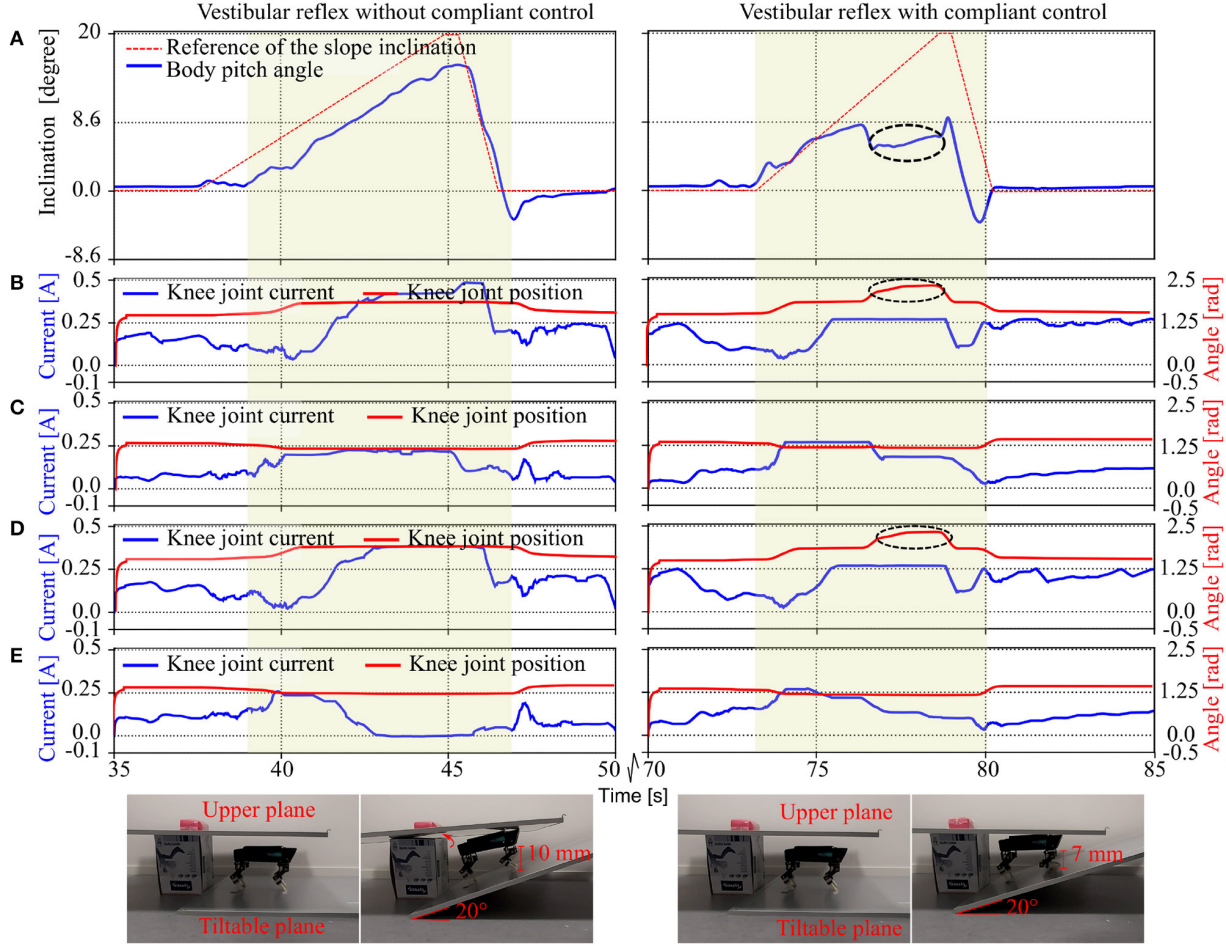
Body stabilization on a tiltable plane with negotiating a payload under vestibular reflex control without and with compliant control. **(A)** The pitch angle of the robot and the reference inclination of the tiltable plane. **(B–E)** are the knee joint positions and currents of the right front (RF), right hind (RH), left front (LF), and left hind (LH) legs, respectively. The yellow colored areas mark the period when the plane was inclined upward. Black circles on the right graphs indicate that the front legs of the robot exhibited compliance to negotiate the payload **(B,D)**. Due to the compliance, the knee joints could flex instead of rigidly resisting the payload, thereby consuming lesser current compared to the case of the pure vestibular reflex control (left graphs). The flexing knee joints could decrease the pitch angle of the robot body [right graph **(A)**], thereby sustaining the body stabilization.

To demonstrate the effectiveness of the combination, we performed two comparative experiments: (1) vestibular reflexes with leg compliance and (2) vestibular reflexes without leg compliance. In both experiments, Lilibot was placed on a tiltable plane under a roof (i.e., an upper plane). The roof acts as a payload (>1.0 kg) if the supported tiltable plane is inclined upward (e.g., 20 degrees) where Lilibot hits the roof. Note that a case with only leg compliance was not used because Lilibot without vestibular reflexes cannot keep balance on the plane if it is tilted or inclined. The experimental results are shown in Figure 12.

It can be seen that the behaviors of the robot under the two controls were different when it negotiated the payload while standing on the slope. Without leg compliance, Lilibot rigidly resisted the payload; thereby, the knee joints of its front legs drew a substantial amount of current (Figures 12B,D). In this situation, the pitch angle of Lilibot also showed a large value (Figure 12A). This could result in imbalance. In contrast, with leg compliance, Lilibot could soften or flex its legs (showing compliance behavior) when it encountered the payload. By doing so, the knee joints of its front legs drew less current (Figures 12B,D) since the Lilibot did not resist the payload. The results indicate that Lilibot under the combination of the vestibular reflex and compliant controls showed better performance and adaptation compared with pure vestibular reflex control. A video clip of this experiment was recorded (at http://www.manoonpong.com/Lilibot/video4.mp4).

## 4. Discussion and Conclusion

In this work, we developed a small size, light weight quadruped robot (Lilibot) with flexible configurations and multiple sensory feedback. Lilibot can act as a friendly open-source platform for research and education in the field of locomotion. The features of small size and light weight provide Lilibot with several apparent advantages, such as an easily modular design, and simple yet practical structure. It can be handled with ease to conduct joint control and locomotion generation owing to its appropriate (1) actuator torque (4.2 Nm, which is not dangerous to handlers), (2) size (its length, width, and height are 30, 17.5, and 20 cm, respectively, when it stands), and (3) weight (2.5 kg) for operation. Moreover, it has a considerable endurance capability, which allows it to handle a payload of ~1.25 kg (50% of its weight) with walking, for up to 30 min. This enables Lilibot to carry extra exteroceptive sensors (e.g., cameras and laser radars for studying motion planning in complex environments). In addition to the real robot, the compatible Lilibot simulation (see Figure 2) allows to develop and test controllers before transferring to the real one.

The experimental results show that Lilibot, with its controller, can exhibit three basic functions, including autonomous gait generation under different reconfigurable leg orientations (Figure 9), compliance behavior for unexpected load compensation (Figure 10), and body stabilization on a tiltable plane (Figure 11). The three functions that we focused on have been found in various animals. They play crucial roles in biological legged locomotion (Dickinson et al., 2000; Fukuoka et al., 2003). The functions are fundamental ingredients for developing an advanced artificial legged system with adaptive, autonomous, and self-organized locomotion. In addition, a variety of sensory feedback (see Table 2) is required to realize the three functions. Therefore, by exploiting these functions, we can effectively demonstrate the capability of Lilibot serving as a quadrupedal platform for research and education in bio-inspired locomotion. We provide the detailed reasons why the three functions are interesting as follows:

Firstly, self-organization of locomotion, in this study, is considered as an ability of a legged system (e.g., Lilibot) that can form a gait in a self-organized manner, in which its inherent physical properties play a crucial role for interlimb coordination via sensory feedback (i.e., continuous via interacting with the ground Owaki et al., 2013. The appropriate single leg movement driven by CPG signals can demonstrate the basic motor function of a leg while the formed interlimb coordination driven by decoupled CPGs with GRF modulations can be used to explore the interaction between robot dynamics and the environment (see Figure 6). The self-organized locomotion realized on the flexible or reconfigurable structures of Lilibot shows both the adaptation of the decoupled CPGs control to its different leg configurations and the utilization of its motor current feedback to reflect the GRF quantity for gait formation. This elucidates the effectiveness of the robot structure design and the used robot actuators with proprioceptive feedback (e.g., current).

In this work, Lilibot shows trot gaits in the four leg configurations under the decoupled CPGs control with the same initialization. The gaits indicate that specific phase relationships among the four CPGs of the legs emerge automatically. The phases of CPGs are inhibited by their continuous GRFs (see Figure 6) if the legs are still on the ground. For example, if a leg is driven to swing by the CPG signals but it cannot swing or lift above the ground, then the GRF will inhibit the CPG signals to make the corresponding leg stay on the ground (stance phase) slightly longer to acquire more GRF. Acquiring more GRF or the maximum GRF during the stance phase of each leg leads to more stable locomotion. A situation that provides maximum GRF at each leg with stable locomotion is when diagonal legs of the robot move at the same phase, e.g., the right front leg and the left hind leg stay on the ground at the same time while the other legs swing in the air and vice versa. This results in a trot gait. This strategy holds for any leg configuration as long as the body can keep balance during a stance phase. An example of the gait generation process can be seen in Figure S3.

In the experiments of the self-organized locomotion (shown in Figure 9), we used a low frequency of the CPGs (i.e., ~0.85 Hz). This is to obtain a slow movement for observing the progression of the phase shifts among the decoupled CPGs during the self-organized process with the predefined frequency; therefore, the robot walked slowly. The obtained gaits were static in all leg configurations because we used “T”-shaped feet, which constantly provide large support areas during walking. However, we can also obtain a dynamic gait by increasing the CPG frequency and using an “O”-shaped feet (see http://www.manoonpong.com/Lilibot/video5.mp4).

Consequently, the self-organization of Lilibot in different leg orientations demonstrates the effectiveness of the robot structure design, the GRF model (see Equations 1–4), and the proprioceptive feedback of joints. It also confirms that Lilibot can easily be used to study the functionality of the limb morphology. However, the flexibly reconfigurable legs are currently organized by a rigid trunk, which cannot be used to study the functionality of the spine dynamics for self-organized locomotion generation. Thus, in the future, we plan to integrate actuated joints in the trunk to connect the front and rear legs, which will imitate a compliant spine with active stiffness.

Secondly, the vestibular reflexes, which are the fundamental biological principle of legged locomotion, have been demonstrated in many quadruped robots for adapting body posture to maintain balance when facing, e.g., an inclination (slope) (Kimura and Fukuoka, 2004; Liu et al., 2013) or a perturbation (Fukui et al., 2019). For instance, when quadrupeds stand or walk on a slope, they need to actively adjust the normal position of their leg joints to acquire proper body posture, thereby sustaining their balance on the slope (as shown in section 3.3 and Fukuoka et al., 2003). In addition, in the work of Fukui et al., the vestibular feedback was used to modulate CPG activities for producing gait transitions (Fukui et al., 2019). The vestibular feedback was integrated into CPG control to improve the adaptation of the interlimb movement pattern that is originally generated by coupled CPGs with predefined connections. However, in our work, the vestibular reflexes were used to directly modulate the outputs of the decoupled CPGs for body posture stabilization (as shown in section 3.3). Our vestibular reflex mechanism and the CPGs control are independent. Thus, one can remove the reflex mechanism without destroying the self-organized locomotion formed by the decoupled CPGs control. Besides, the achievement of the vestibular reflexes can illustrate the effectiveness of the controlled structure (i.e., Lilibot structure) and the vestibular feedback.

Thirdly, compliance is a vital characteristic of muscles. It allows biological and artificial legged systems to rapidly adapt to external disturbances [such as an unexpected load compensation (as shown in section 3.2) and uneven terrain locomotion (Xiong et al., 2015)]. Thus, implementing compliance can prevent the robot from being damaged by the disturbance. Moreover, the compliant control can cooperate with vestibular reflex control to realize greater body stabilization when facing a payload on a slope (as shown in section 3.4) and allow for energy efficient locomotion when walking on uneven terrains (Xiong et al., 2015).

Taken together, the self-organization allows a quadruped robot to automatically form adaptive gaits, whereas the vestibular reflexes enable the robot to maintain balance on a non-level ground or slope and the joint compliance can prevent damage as well as lead to energy efficient locomotion (Xiong et al., 2015). A combination of the three functions will be performed in the future as one of our research plans.

In addition to the discussions of the three functions, we review the foot structure of Lilibot here. In contrast to the general foot shapes used previously, the leg structure developed and employed here, with the “T”-shaped feet (see Figure 3C) significantly increases the walking stabilization. This is because the “T”-shaped feet provide a higher static stability margin compared to other foot shapes, such as the ball-shaped foot used by Oncilla (Sproewitz et al., 2011) and the half-cylinder-shaped foot used by Tekken (Kimura and Fukuoka, 2004). The “T”-shaped feet allow users to focus on the interlimb coordination of the gait generations, and hence, overcome the problems of intralimb coordination for improving stabilization. However, this shape is not beneficial for lateral stepping due to the smaller lateral contact area, and it is also challenging to adapt to uneven terrain. Therefore, we plan to develop new adaptive compliant feet with a relatively high stability margin and contact area (Canio et al., 2016; Hauser et al., 2018).

In summary, we have successfully developed a small-sized and lightweight quadruped robot, known as Lilibot. The structure of Lilibot, which imitates four-limbed mammals, such as dogs, consists of four identical legs connected by a rigid trunk, as well as “T”-shaped feet with large support areas to provide a higher static stability margin. Each leg has only three active DOFs. Nevertheless, the large joint workspace enables the robot to exhibit flexible leg orientations to imitate various types of mammal morphologies. This characteristic of the robot contributes to studying the adaptation of self-organized locomotion regarding various leg configurations, based on different biological systems (for example, dogs, horses, and infants). This advantage was demonstrated by using decoupled CPGs to control Lilibot under its four leg orientation types in the experiments. Moreover, inspired by a hexapod robot (Mathias et al., 2018), the suitable smart actuators on the joints are employed, which not only simplify the electric system of the robot, but also provide a large variety of sensory feedback (61 sensory feedback signals in total). The sensory feedback allows Lilibot to perform compliant and vestibular reflex controls, thereby demonstrating external load negotiation and body stabilization, respectively. Based on the results, we suggest that Lilibot can be considered as a friendly and generic quadrupedal platform for studying self-organized locomotion, vestibular reflexes, and compliant behavior.

## Data Availability Statement

All datasets generated for this study are included in the article/Supplementary Material.

## Author Contributions

TS developed the entire robotic system, performed the experiments, analyzed the data, and drafted the manuscript. XX led the development of compliant control and reviewed the manuscript. ZD provided the general direction of the project and reviewed the manuscript. PM led the development of adaptive neural control, helped with data analysis, drafted the manuscript, and supervised the whole project. All authors contributed to the conception and design of the work, approved the final version of the manuscript, and agree to be accountable for all aspects of the work.

### Conflict of Interest

The authors declare that the research was conducted in the absence of any commercial or financial relationships that could be construed as a potential conflict of interest.

## Footnotes

^1^ https://www.bostondynamics.com/ls3

^2^ https://www.bostondynamics.com/wildcat

^3^ https://www.bostondynamics.com/spot-classic

^4^ https://www.unitree.cc

^5^ http://www.robotis.us/

^6^ http://www.manoonpong.com/Lilibot/video0.mp4

^7^In this paper, we briefly describe the controller since it is not the main focus of the paper, but it is necessary for demonstrating the performance of our open-source platform Lilibot (which is our main focus).
