# Corrigendum: Small-Sized Reconfigurable Quadruped Robot With Multiple Sensory Feedback for Studying Adaptive and Versatile Behaviors

**DOI:** 10.3389/fnbot.2021.746056

**Published:** 2021-08-13

**Authors:** Tao Sun, Xiaofeng Xiong, Zhendong Dai, Poramate Manoonpong

**Affiliations:** ^1^Institute of Bio-inspired Structure and Surface Engineering, College of Mechanical and Electrical Engineering, Nanjing University of Aeronautics and Astronautics, Nanjing, China; ^2^Embodied AI & Neurorobotics Lab, SDU Biorobotics, Mærsk Mc-Kinney Møller Institute, University of Southern Denmark, Odense, Denmark

**Keywords:** quadruped robot, multiple sensory feedback, self-organized locomotion, vestibular reflexes, compliant control, flexible configuration

In the published article, there was a spelling error in affiliation 2. Instead of “Embodied AI & Neurobotics Lab, SDU Biorobotics, Mærsk Mc-Kinney Møller Institute, University of Southern Denmark, Odense, Denmark,” it should be “Embodied AI & Neurorobotics Lab, SDU Biorobotics, Mærsk Mc-Kinney Møller Institute, University of Southern Denmark, Odense, Denmark.”

The authors apologize for this error and state that this does not change the scientific conclusions of the article in any way. The original article has been updated.

## Publisher's Note

All claims expressed in this article are solely those of the authors and do not necessarily represent those of their affiliated organizations, or those of the publisher, the editors and the reviewers. Any product that may be evaluated in this article, or claim that may be made by its manufacturer, is not guaranteed or endorsed by the publisher.

