# Supplementary material for: Small-Sized Reconfigurable Quadruped Robot With Multiple Sensory Feedback for Studying Adaptive and Versatile Behaviors
Source: Front Neurorobot. 2020 Feb 26;14:14. doi: 10.3389/fnbot.2020.00014 (PMC7054281; doi:10.3389/fnbot.2020.00014)
Supplement: Supplementary file 1 [file Data_Sheet_1.PDF]

Supplementary Material

1 SUPPLEMENTARY FIGURES

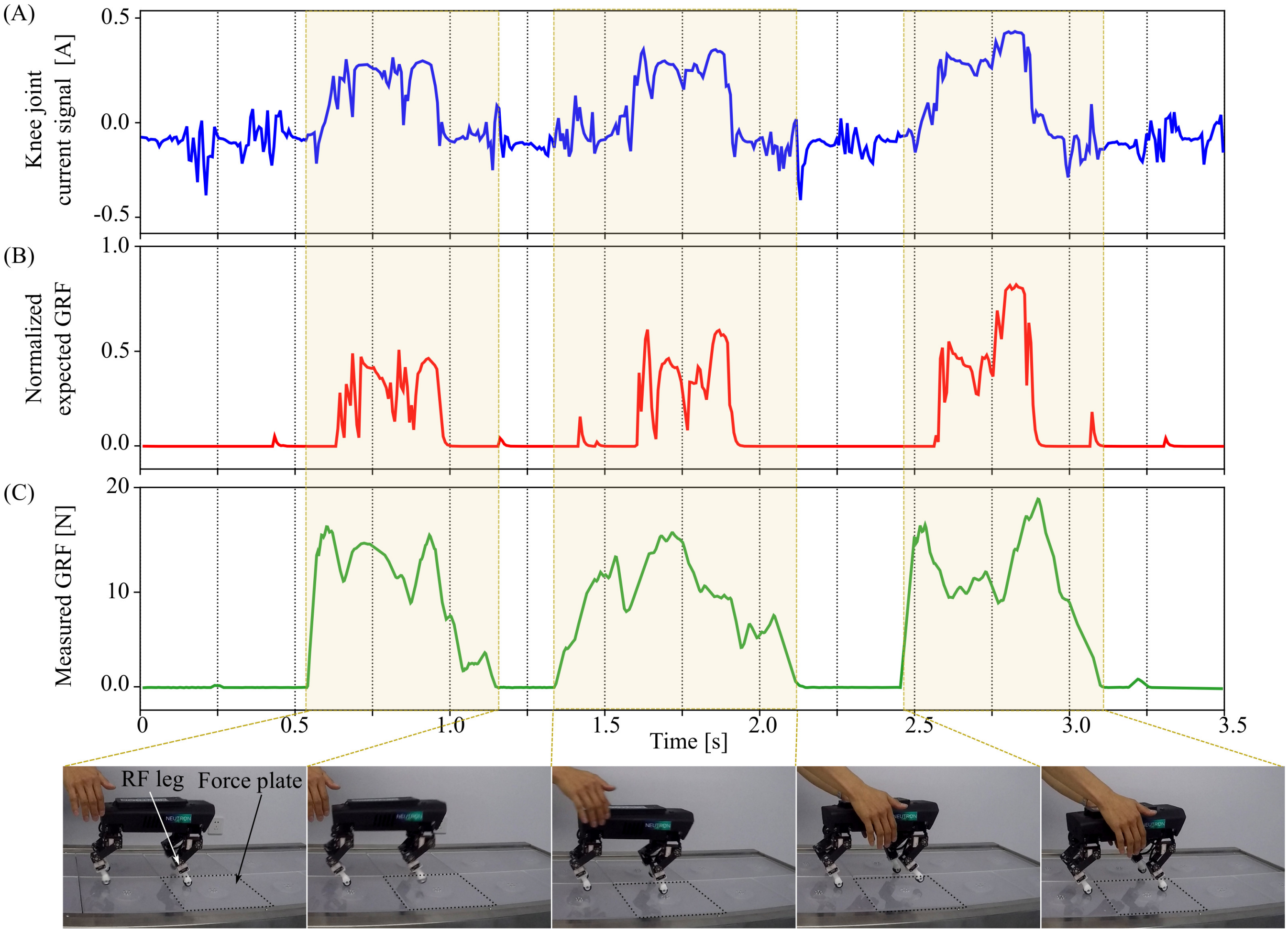

Figure S1. Experimental process for tuning the parameters of the GRF model (see Eqs. 1-4 in the main manuscript). The model translates the knee joint current signal (A) to the normalized expected ground reaction force (GRF) (B). The normalized GRF is in a range (0, 1) which is then used for our decoupled CPGs control. The measured GRF (obtaining from the custom-designed force plate platform for legged robots) is used as a baseline for tuning the model parameters. One can observe a positive correlation between the keen joint current signal and the GRF signal. The signals show high activation (> 0.0) when the leg is in a stance phase (color area) and low activation (around 0.0) when it is in a swing phase (white area). In this graph, we show an example of the signals of the right front leg.

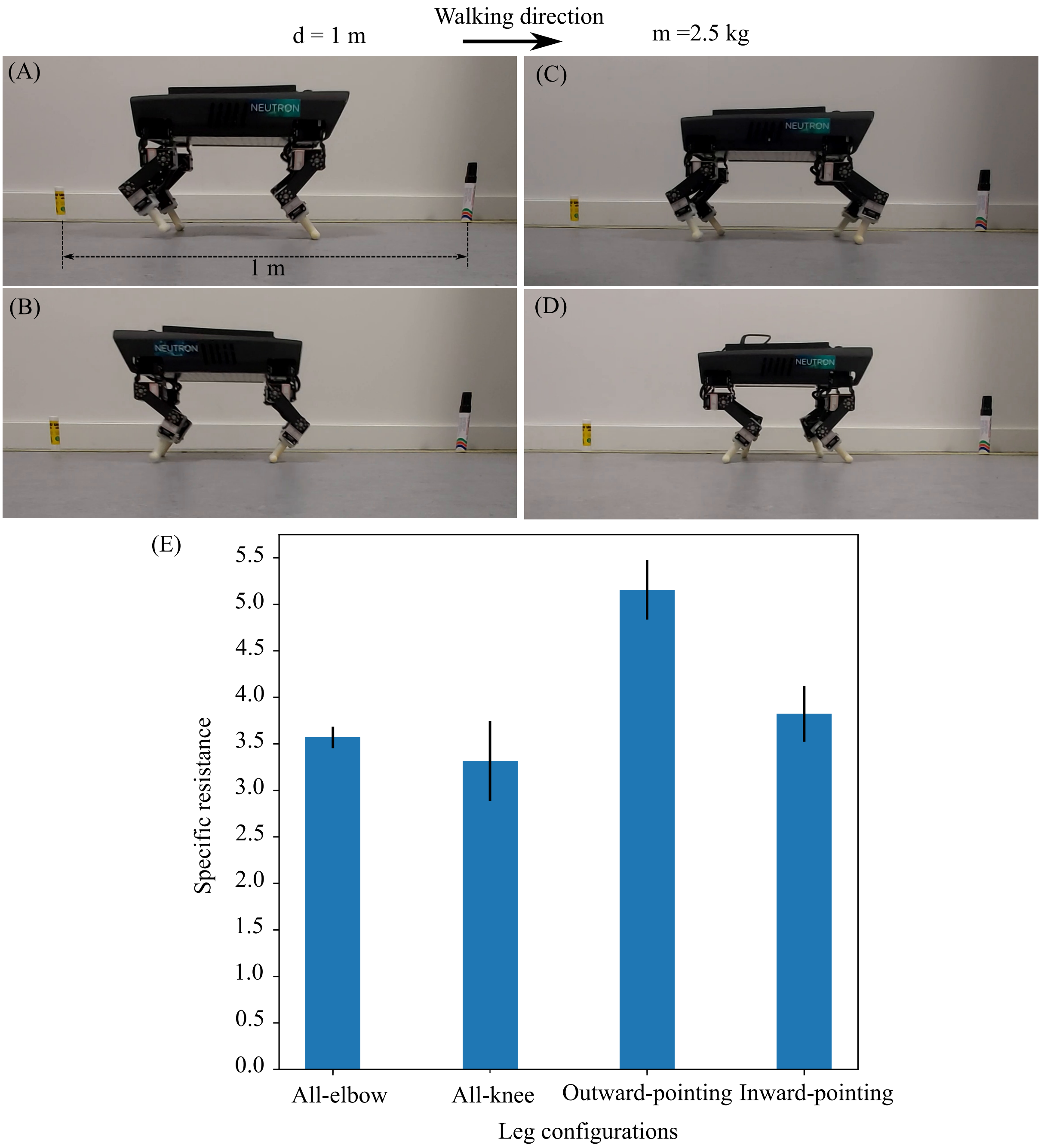

Figure S2. Walking experiments for measuring the specific resistances of Lilibot under four leg configurations: (A) All-elbow, (B) All-knee, (C) Outward-pointing, and (D) Inward-pointing. For each leg configuration, the robot was set to walk for one meter in a straight line for five times. The robot was driven with the same controller (i.e., same CPG frequency, around 2.2 Hz). The power consumption was recorded during the experiments to calculate the specific resistances. The specific resistance (ϵ, see Eq. 5 in the main manuscript) also known as the cost of transport (COT) is used to demonstrate the energy-efficiency of locomotion. Low ϵ corresponds to high energy-efficient walking. (E) The specific resistance of the robot walking in the experiments. The results show that the all-knee configuration leads to the most energy-efficient locomotion.

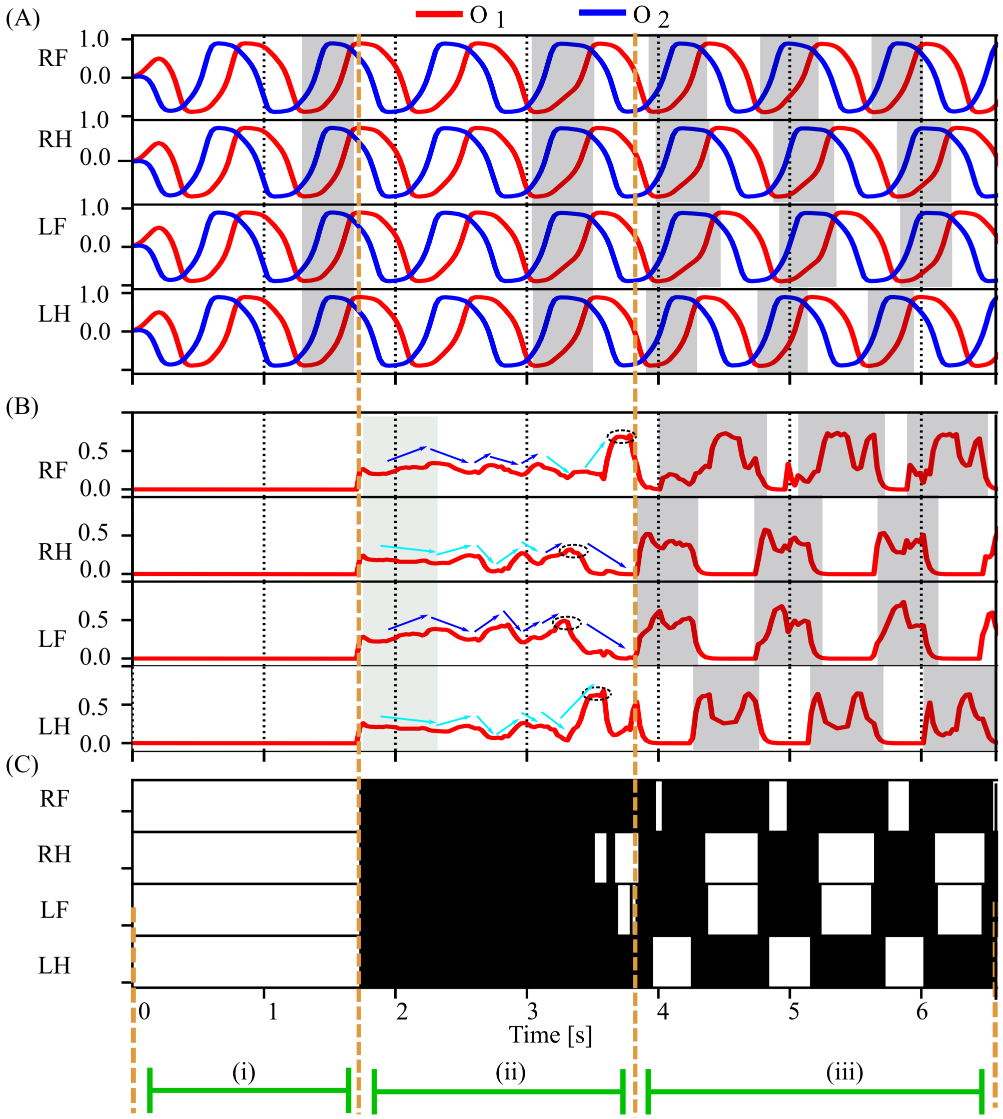

Figure S3. Mechanism of the gait generation process under decoupled CPGs control with GRFs modulations or inhibitions. (A) CPG outputs of the four legs. (B) Normalized GRFs of the four legs. The color arrows indicate the changing tendency of the GRFs. The black circles point out the leg's peak force at (ii) stage. (C) Gait diagram. Black and white areas indicate stance and swing phases, respectively. The gait generation process includes three stages: (i) Four decoupled CPGs with the same phase initialization where the robot was held in the air (GRFs were zero), (ii) Progression of the phase shifts among the four CPGs while the CPGs interact with the environment through GRFs feedback where the robot was placed on the ground, and (iii) A trot gait emerged. Once the robot was placed on the ground at (ii) stage, all legs tended to stay at the stance phase to competitively acquire more GRF to inhibit their corresponding CPG (see the decoupled CPGs model in the next subsection). The front legs (RF and LF) or hind legs (RH and LH) had the same movement at the beginning (light gray area in (ii)). Once diagonal legs (i.e., RH and LF legs) had the same movement, they can acquire their own largest GRFs where the diagonal legs were able to stably support the robot. This results in the other diagonal legs (i.e., RF and LH legs) had small GRFs. On the next step, the diagonal legs (i.e., RF and LH legs) moved to a stance phase and got more GRFs. At the same time, the other diagonal legs (i.e., RH and LF legs) switched to a swing phase. During this progression, a trot gait finally emerged in stage (iii).

2 SUPPLEMENTARY MODEL

The decoupled CPGs can be described by following discrete-time equations:

$$a_1^k(n+1) = \sum_{j=1}^{2} w_{1j} o_j^k(n) + B_1^k - \gamma * f(n)^k * cos(o_1^k), k = 1, \cdots, 4 \tag{S1}$$

$$a_2^k(n+1) = \sum_{j=1}^{2} w_{2j} o_j^k(n) + B_2^k - \gamma * f(n)^k * sin(o_2^k), k = 1, \cdots, 4 \tag{S2}$$

$$o_i^k(n) = \tanh(a_i^k(n)), i = 1, 2, k = 1, \cdots, 4 \tag{S3}$$

where $a_{1,2}^k$ and $o_{1,2}^k$ are the activities and outputs of the CPG k. $B_{1,2}^k$ are the internal biases of the CPG k. The last terms on the right side of Eqs. S1 and S2 are the GRF inhibitions to the CPGs. f^k is the GRF feedback of the leg k. The sum of the $f^k(n)$ provides a function to supprot the weight mg of the robot. It suggests that there is a physical communication between the four CPGs through GRFs feedback. The communication is characterized by the robot rigid body.
